# Supplementary material for: A combination of phenotypic responses and genetic adaptations enables Staphylococcus aureus to withstand inhibitory molecules secreted by Pseudomonas aeruginosa
Source: mSystems. 2025 Jul 31;10(8):e00262-25. doi: 10.1128/msystems.00262-25 (PMC12363241; doi:10.1128/msystems.00262-25)
Supplement: Supplemental material — Figures S1 to S3; Tables S1 to S5. [file msystems.00262-25-s0001.pdf]

Supplemental Material

A combination of phenotypic responses and genetic adaptations enables *Staphylococcus aureus* to withstand inhibitory molecules secreted by *Pseudomonas aeruginosa*

Lukas Schwyter[a], Selina Niggli[a], Natalia Zajac[b,c], Lucy Poveda[b,c], Witold Eryk Wolski[b], Christian Panse[b,c], Ralph Schlapbach[b], Rolf Kümmerli[a,*], Jonas Grossmann[b,c,*]

[a] Department of Quantitative Biomedicine, University of Zurich, Winterthurerstrasse 190, 8057 Zürich, Switzerland

[b] Functional Genomics Center Zurich, ETH Zurich and University of Zurich, 8057 Zurich, Switzerland

[c] Swiss Institute of Bioinformatics (SIB) Quartier Sorge - Batiment Amphipole, 1015 Lausanne, Switzerland

* equal contribution

Corresponding authors

Jonas Grossmann, jonas.grossmann@fgcz.uzh.ch

Rolf Kümmerli, rolf.kuemmerli@uzh.ch

This document contains the following supplementary material:

- 3 supplemental Figures
- 5 supplemental Tables

Supplemental Figure S1

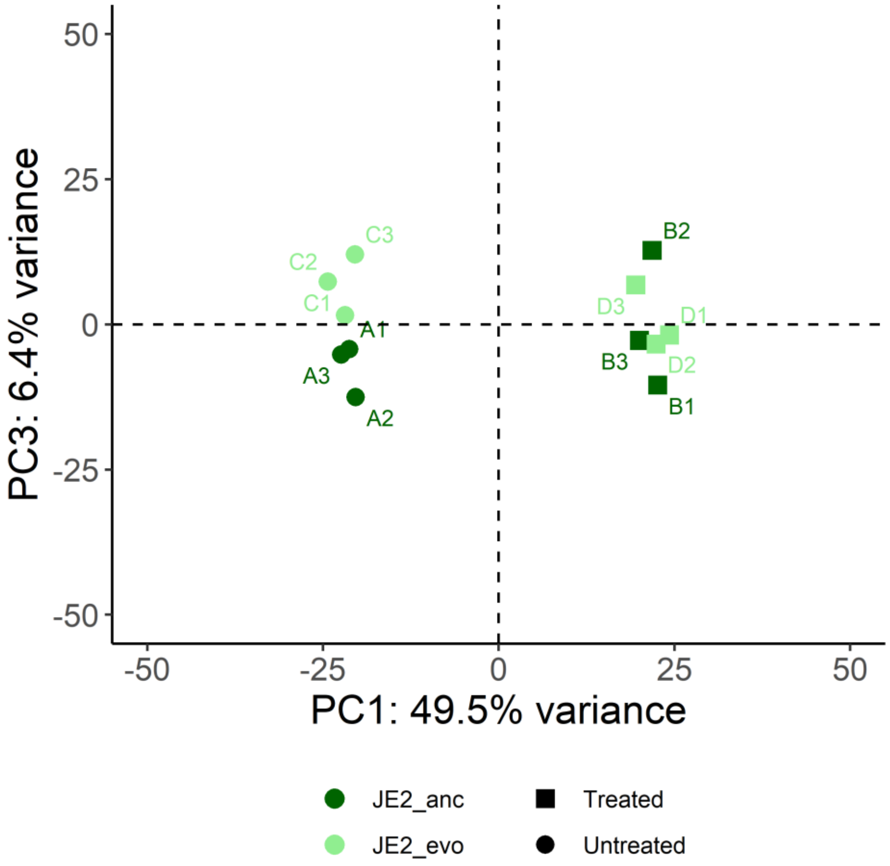

Figure S1. Principle component (PC) analysis on all measured proteins showing the first and third PC. They account together for 55.9% variation observed in the data. Squares and circles differentiate replicates grown in either 70% tryptic soy broth (TSB) supplemented with 30% spent supernatant of *P. aeruginosa* PAO1 (square) or 100% TSB medium (circle). Light green symbols: JE2_evo. Dark green symbols: JE2_anc.

Supplemental Figure S2

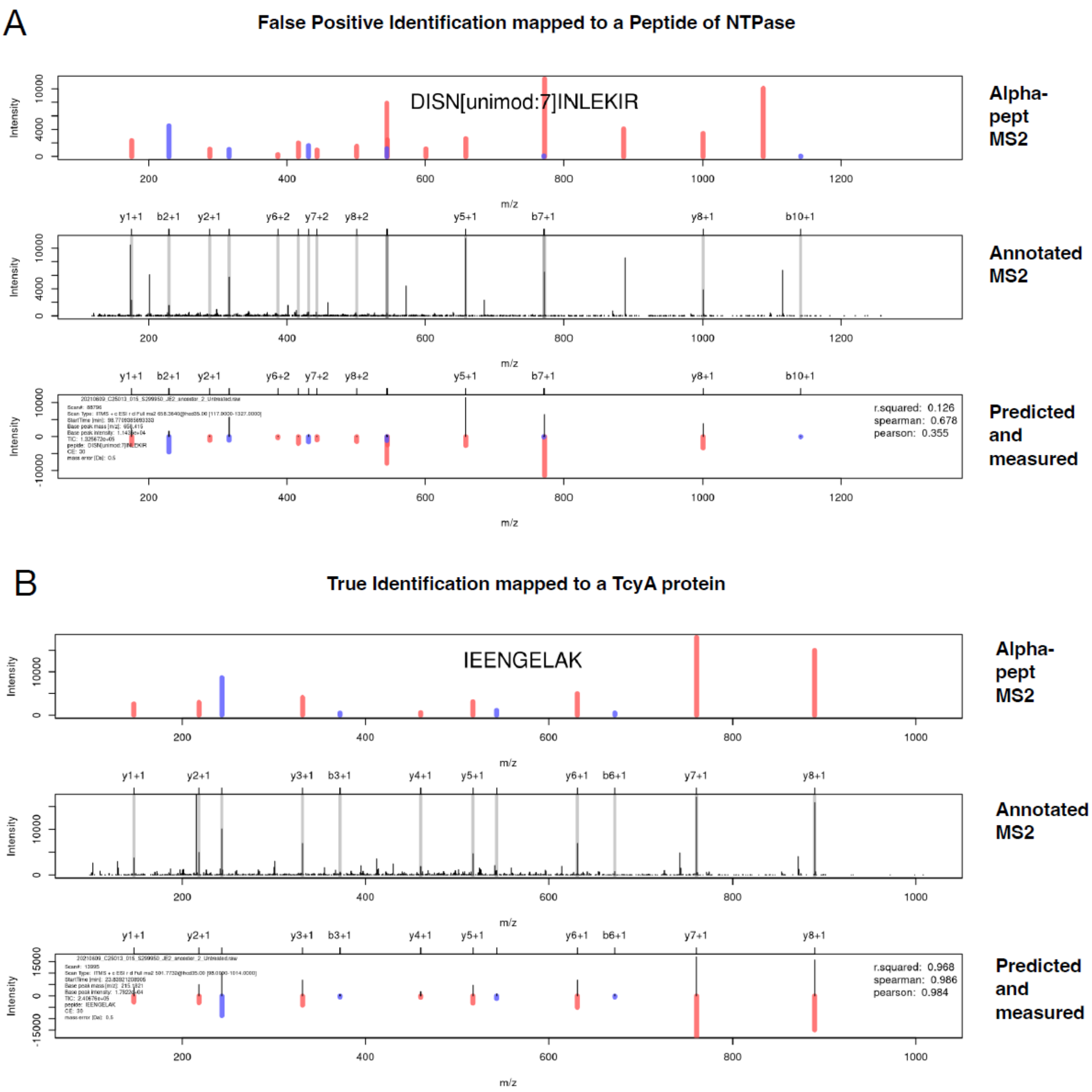

Figure S2. To illustrate the false positive match of the DISN[unimod:7] INLEKIR peptide, we predicted the spectrum using the AlphaPept_ms2_generic model, which is available through the Koina model repository. As an initial step, we conducted a collision energy (CE) normalization using true positive (TP) hits, such as the peptide IEE shown in panel B. The resulting CE value of 30 eV was then used as input together with the peptide sequence and the charge state to predict the DISNI peptide spectrum (Gessulat et al., 2019; Zeng et al., 2022). Panel A displays the evidence for the identification of the NTPase protein, which was determined to be a false positive. Panel B shows a valid identification for a peptide from the TcyA protein. In both panels, the top section illustrates the predicted y-ion (red) and b-ion (blue) spectra generated by koina (Alpha-pept MS2). The middle section presents the experimentally measured and annotated spectrum with matched y- and b-ions (Annotated MS2). The bottom section features a mirror plot comparing the experimental and predicted spectra for the matched ions (Predicted and Measured).This allows to calculate correlation statistics to identify true from false positive identifications.

Supplemental Figure S3

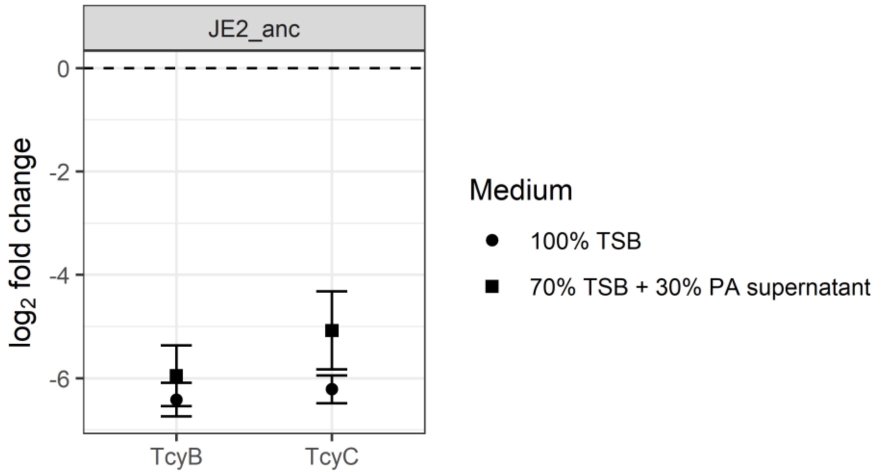

Figure S3. Log-fold intensity change of TcyB and TcyC relative to TcyA for JE2_anc, grown in either 100% TSB or 70% TSB + 30% PA supernatant. The analysis shows that TcyA is much more abundant than TcyB and TcyC, indicating that TcyABC is a Type II ATP-Binding Cassette transporter, where the substrate binding protein TcyA occurs in high excess and does not directly bind to the permease TcyB.

Supplemental Table S1 KEGG Pathways

Pathway	Total Proteins	Non-Significant regulated	Significant upregulated	Significant downregulated	BH-adjusted p-value
6. Human Disease	54	29	2	23	0.0020
3. Environmental Information Processing	142	96	13	33	0.0461
3.2 Signal transduction	72	48	7	17	0.1600
4. Cellular Processes	26	16	1	9	0.2200
3.1 Membrane transport	70	48	6	16	0.2200
1.1 Carbohydrate metabolism	272	201	41	30	0.3310
1.5 Amino acid metabolism	163	121	20	23	0.4440
1.6 Metabolism of other amino acids	31	23	5	3	0.8390
1.2 Energy metabolism	57	43	8	6	0.8390
1.8 Metabolism of cofactors and vitamins	94	74	14	6	1
1.7 Glycan biosynthesis and metabolism	31	25	0	6	1
DUF	91	73	5	13	1
NA	765	599	66	100	1
1.3 Lipid metabolism	57	48	4	5	1
1.4 Nucleotide metabolism	104	86	6	12	1
1.9 Metabolism of terpenoids and polyketides	28	26	1	1	1
2. Genetic Information Processing	171	159	7	5	1
Enzymatic Reaction	638	496	71	71	Not a KEGG pathway

Supplemental Table S2 Proteins from the human disease pathway (KEGG annotation), grouped according to function based on the virulence factor database (VFDB)

Protein	Fold change (log2)[a]	BioCyc Annotation	VFDB annotation
HlgA	2.65	0.0002	Exotoxin
EssC	1.96	0.3237	Effector delivery system
Rot	1.70	0.0018	Quorum Sensing
SecA	0.28	0.2795	Exotoxin
IsdA	0.27	0.4787	Nutritional/Metabolic factor
IsdI	0.27	0.6112	Nutritional/Metabolic factor
Spa	0.27	0.5415	Exotoxin
Ssl3	0	1.0000	Exotoxin
AgrB	-0.07	0.8980	Quorum Sensing
Ssl10	-0.11	0.9722	Exotoxin
SecA2	-0.19	0.7812	Exotoxin
ScpB	-0.22	0.8793	Exoenzyme
Scn	-0.34	0.6984	Immune modulation
SecY	-0.35	0.5932	Exotoxin
EbpS	-0.41	0.2888	Adherence
EsxA	-0.46	0.3823	Effector delivery system
FnbB	-0.59	0.5023	Adherence
Ssl14	-0.67	0.2231	Exotoxin
SdrD	-0.69	0.0381	Adherence
SecDF	-0.77	0.0349	Exotoxin
ClfB	-0.83	0.0225	Adherence
EsaG	-0.88	0.3652	Effector delivery system
IsdB	-1.01	0.8062	Nutritional/Metabolic factor
EssB	-1.02	0.6817	Effector delivery system
ToxC	-1.03	0.4250	Exotoxin
AgrC	-1.03	0.0344	Quorum Sensing
IcaR	-1.06	0.0039	Adherence
AgrA	-1.32	0.0006	Quorum Sensing
GehE	-1.36	0.0840	Exoenzyme
SdrH	-1.51	0.0298	Adherence
Cap1C	-1.53	0.4938	Immune modulation
HlgB	-1.55	0.0205	Exotoxin
FnbA	-1.55	0.4144	Adherence
AdsA	-1.74	0.0015	Immune modulation
IsdH	-2.09	0.3061	Nutritional/Metabolic factor
ToxC	-2.22	0.0023	Exotoxin
GehB	-2.23	0.0000	Exoenzyme
GehA	-2.83	0.0002	Exoenzyme
SspB	-3.16	0.4627	Exoenzyme
EsaA	-3.25	0.0017	Effector delivery system
ClfA	-3.68	0.0000	Adherence
HlgC	-3.70	0.0075	Exotoxin
Ssl6	-3.72	0.3094	Exotoxin
SdrC	-3.76	0.0260	Adherence
Eap/Map	-4.02	0.0000	Adherence

Sbi	-4.33	0.0000	Immune modulation
Hla	-5.08	0.0000	Exotoxin
LukG	-5.09	0.0000	Exotoxin
LukD	-5.16	0.0144	Exotoxin
LukH	-5.39	0.0000	Exotoxin
ScpA	-6.04	0.0000	Exoenzyme

Supplemental Table S3 Transporter genes differently expressed in *S. aureus* grown in PAO1 supernatant vs TSB

Protein	Fold change (log2)[a]	BioCyc Annotation	Primary Function
EmrB	5.04	MFS transporter	Exporter
GntP	4.43	Gluconate permease	Importer
BrnQ1	3.70	Branched-chain amino acid transporter	Importer
GlvC		PTS alpha-glucoside transporter subunit	Importer
	2.83	IIBC	Importer
Q2G144_STAA8	1.65	PTS lactose transporter subunit IIB	
TreP	1.35	PTS trehalose transporter subunit IIBC	Importer
HtsA	1.19	Heme ABC transporter substrate-binding protein	Importer
MntA	1.04	Phosphonate ABC transporter ATP-binding protein	Importer
CntA	-1.05	Nickel ABC transporter	Importer
Q2FVB4_STAA8	-1.05	Lantibiotic ABC transporter ATP-binding protein	Importer
SirA	-1.27	Iron ABC transporter substrate-binding protein	Importer
Rnd2	-1.31	Multidrug transporter	Exporter
PotD	-1.36	Spermidine ABC transporter substrate-binding protein	Importer
MetN	-1.51	ABC transporter ATP-binding protein	Importer
Q2FVG0_STAA8	-1.54	ABC transporter ATP-binding protein	Importer
MurP	-1.72	Permease	Importer
DltD	-1.73	D-alanyl-lipoteichoic acid biosynthesis protein	Not categorized
Q2FYU8_STAA8	-1.76	Gamma-aminobutyrate permease	Importer
OpuBB	-1.83	Glycine/betaine ABC transporter permease	Importer
NsaA	-1.98	ABC transporter ATP-binding protein	Exporter
Opp3F	-1.99	Oligopeptide ABC transporter ATP-binding protein	Importer
YvrC	-2.02	ABC transporter substrate-binding protein	Importer
Opp3D	-2.08	ABC transporter ATP-binding protein	Importer
GmpC	-2.35	ABC transporter substrate-binding protein	Importer
NptA	-2.36	Na/Pi cotransporter	Importer
MscL	-2.89	Mechanosensitive ion channel protein MscL	Not categorized
PhnD	-3.49	Phosphonate ABC transporter substrate-binding protein	Importer
CrtQ	-3.88	4,4-diaponeurosporenoate glycosyltransferase	Not categorized
Q2G1F5_STAA8	-4.70	Predicted ABC transporter of a peptide	Importer
DacA	-4.71	TIGR00159 family protein	Not categorized
Q2FZM4_STAA8	-4.95	ABC transporter substrate-binding protein	Importer

Opp4A	-5.08	Nickel ABC transporter substrate-binding protein	Importer
Opp3A	-5.16	Peptide ABC transporter substrate-binding protein	Importer
EpiF	-5.95	Lantibiotic ABC transporter ATP-binding protein	Exporter
SAUPAN001056000 potential orthologue OppF	MS/MS evidence that protein is only expressed in untreated condition. Peptide intensity is weak but can consistently only be found in untreated conditions		Importer

[a] Fold change values are the average of 3 replicates after normalization

Supplemental Table S4 Strains used for this study.

Species and strain name	Origin	Description	Reference
Pseudomonas aeruginosa (PA)			
PAO1	Wound	Commonly used *Pseudomonas aeruginosa* laboratory strain.	ATCC 15692
Staphylococcus aureus (SA)			
JE2_anc	Skin and soft tissue infection	USA300 CA-MRSA isolate. Highly virulent, cytotoxic, and hemolytic.	NARSA
JE2_evo	Skin and soft tissue infection	USA300 CA-MRSA isolate. Highly virulent, cytotoxic, and hemolytic, Framshift mutation in the *tcyABC* operon	Niggli et al. 2023 (B0604)
JE2 *tcyA*::Tn	Skin and soft tissue infection	USA300 CA-MRSA isolate. Highly virulent, cytotoxic, and hemolytic, Tn insertion in the *tcyA* gene	Nebraska transposon mutant library
JE2 *emrB*::Tn	Skin and soft tissue infection	USA300 CA-MRSA isolate. Highly virulent, cytotoxic, and hemolytic, Tn insertion in the *emrB* gene	Nebraska transposon mutant library

CA-MRSA: Community-acquired methicillin-resistant *S. aureus*

Supplemental Table S5 Primers used for amplicon sequencing

Target Gene	Encoded Protein	Sequence	Amplicon (bp)
tcyA	Amino acid ABC transporter substrate-binding protein	F:5-GCATGTTGTTGTTCGCTATT-3	333
		R:5-GCTAAGGATAAAGGTGCTGA-3	
